# The Effect of Preparation Conditions on Raman and Photoluminescence of Monolayer WS_2_

**DOI:** 10.1038/srep35154

**Published:** 2016-10-18

**Authors:** Kathleen M. McCreary, Aubrey T. Hanbicki, Simranjeet Singh, Roland K. Kawakami, Glenn G. Jernigan, Masa Ishigami, Amy Ng, Todd H. Brintlinger, Rhonda M. Stroud, Berend T. Jonker

**Affiliations:** 1 Naval Research Laboratory, Washington DC 20375, USA; 2 Department of Physics, The Ohio State University, Columbus OH 43210, USA; 3 Department of Physics and Nanoscience Technology Center, University of Central Florida, Orlando, FL 32816-2385, USA

## Abstract

We report on preparation dependent properties observed in monolayer WS_2_ samples synthesized via chemical vapor deposition (CVD) on a variety of common substrates (Si/SiO_2_, sapphire, fused silica) as well as samples that were transferred from the growth substrate onto a new substrate. The as-grown CVD materials (as-WS_2_) exhibit distinctly different optical properties than transferred WS_2_ (x-WS_2_). In the case of CVD growth on Si/SiO_2_, following transfer to fresh Si/SiO_2_ there is a ~50 meV shift of the ground state exciton to higher emission energy in both photoluminescence emission and optical reflection. This shift is indicative of a reduction in tensile strain by ~0.25%. Additionally, the excitonic state in x-WS_2_ is easily modulated between neutral and charged exciton by exposure to moderate laser power, while such optical control is absent in as-WS_2_ for all growth substrates investigated. Finally, we observe dramatically different laser power-dependent behavior for as-grown and transferred WS_2_. These results demonstrate a strong sensitivity to sample preparation that is important for both a fundamental understanding of these novel materials as well as reliable reproduction of device properties.

The novel properties of graphene have stimulated research in other two-dimensional (2D) materials such as hexagonal boron nitride and the transition metal dichalcogenides (TMDs). The TMDs have a chemical composition of MX_2_ (where M = Mo, W and X = S, Se), and monolayer building blocks of these materials are composed of three sheets of atoms where a top and bottom chalcogen layer encapsulate the center metal sheet. While much of the early work focused on monolayer MoS_2_, the closely related WS_2_ exhibits superior optical properties^1^,^2^,^3^ and larger spin-orbit coupling^4^, motivating further extensive investigation. WS_2_ exhibits a strong sensitivity to layer number, with the band structure transitioning from an indirect-gap semiconductor for bulk WS_2_ to direct-gap semiconductor at monolayer thickness^1^. Fundamental investigations and prototype devices incorporating atomically thin WS_2_ have demonstrated reasonable electronic mobility^5^,^6^, high optical responsivity^1^, robust device performance following repeated bending^7^, high valley polarization^8^,^9^,^10^, and long spin lifetimes^11^,^12^. These properties make WS_2_ a promising material for a variety of applications including photodetection, flexible electronics, and spintronics.

Monolayers of TMDs can be obtained using a variety of methods including mechanical and chemical exfoliation, atomic layer deposition (ALD)^13^, molecular beam epitaxy (MBE)^14^, and chemical vapor deposition (CVD)^15^. Other innovative options continue to be explored. The CVD approach has emerged as a particularly reliable route to obtain uniform, high-quality, large-area samples^2^,^16^,^17^, with wafer-scale synthesis of WS_2_ recently demonstrated^18^. Due to the elevated temperatures necessary for CVD synthesis, rigid and thermally robust substrates such as SiO_2_ or Al_2_O_3_ are commonly used.

The ability to transfer films off the growth substrate provides a means to incorporate TMDs with a more diverse array of materials, and is highly desirable for both fundamental investigations and applications. For instance, transfer to high-k dielectrics may provide a means to modify excitonic properties such as binding energies, position, and radii, as Coulomb interactions are sensitive to the local environment^19^,^20^. The construction of van der Waals heterostructures (vdwh) through sequential stacking of 2D materials is also expected to open the door to new device functionality, and is a rapidly progressing area of research^21^ with recent reports of interlayer excitons^22^, ultrafast charge separation^23^, and long valley lifetimes^24^ in TMD based vdwh. While reliable transfer techniques will certainly be important as the field continues to grow, there are limited reports that investigate if and how WS_2_ is impacted^25^,^26^,^27^. Additionally, such studies typically transfer between dissimilar materials (i.e. sapphire to Si/SiO_2_), making it difficult to determine the source of any observed modifications. Therefore, it is important to first understand and evaluate any changes brought about by transfer processes between identical substrates so that future investigations may accurately isolate, identify and assess modifications resulting from interactions with various materials. In this letter we directly compare fundamental properties of CVD synthesized monolayer WS_2_ between the as-grown state (as-WS_2_) and after removal from the growth substrate and transfer to a fresh supporting substrate (x-WS_2_).

## Results and Discussion

**Figure 1:**
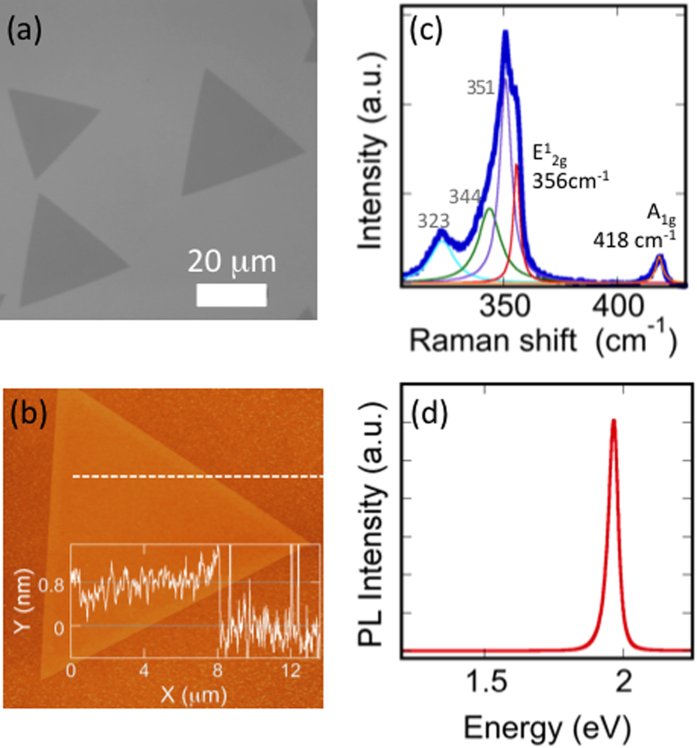
Characterization of monolayer WS_2_ synthesized on Si/SiO_2_. (**a**) Optical microscope images display randomly oriented equilateral triangular growth. (**b**) The AFM image is obtained on a representative WS_2_ triangle. A height profile (inset) is measured along the dashed line and shows a step height of ~0.8 nm. (**c**) Raman spectroscopy and (**d**) photoluminescence measurements confirm monolayer WS_2_.

Monolayer tungsten disulfide is synthesized at a growth temperature of 825 **°**C in a 2-inch diameter quartz tube furnace. Powdered WO_3_ (Alfa Aesar 13398) and sulfur (Alfa Aesar 10755) serve as precursors for the synthesis. Additional details can be found in reference^16^. Typically Si/SiO_2_ (275 nm) is employed as growth substrate and is the main focus of this manuscript. However, monolayer WS_2_ has also been realized on c-plane sapphire and fused silica substrates, and will be briefly discussed. Growth on Si/SiO_2_ results in multiple triangular islands exhibiting lateral dimensions ranging from a few to several tens of microns, as shown in the optical image, Fig. 1(a). The equilateral triangle morphology is characteristic of single-crystal growth^28^. Examination of a WS_2_ triangle using AFM (Fig. 1(b)) shows uniform thickness across the triangle as well as a step height of ~0.8 nm (inset of Fig. 1(b)), consistent with monolayer WS_2_.

Raman and photoluminescence (PL) spectroscopy measurements are performed using a commercial Horiba LabRam confocal spectrometer in ambient atmosphere at room temperature using 532 nm laser excitation unless otherwise stated. A 100× objective focuses the laser beam to a spot with ~1 μm diameter. Raman spectroscopy is a quick and reliable method for layer identification in WS_2_^29^,^30^. In addition to the in-plane (E^1^_2g_) and out-of-plane (A^1^_g_) first order optical modes, 532 nm laser excitation results in second-order resonant peaks involving longitudinal acoustic modes (LA(M)). A typical Raman spectrum from as-WS_2_ is show in Fig. 1(c). A multi-lorentzian fit clearly resolves the individual components, with E^1^_2g_ and A^1^_g_ peaks located at 355.7 cm^−1^ and 418.4 cm^−1^, respectively. A PL spectrum (Fig. 1(d)), also measured on as-WS_2_, shows a single peak having maximum intensity at ~1.96 eV, confirming the single layer nature. While sample-to-sample variation can influence the exact PL peak position, repeated measurements obtained across multiple samples as well as separate monolayer regions on the same sample typically exhibit peak emission intensity within ~10 meV of 1.96 eV for monolayer WS_2_ synthesized on Si/SiO_2_ (275 nm).

**Figure 2:**
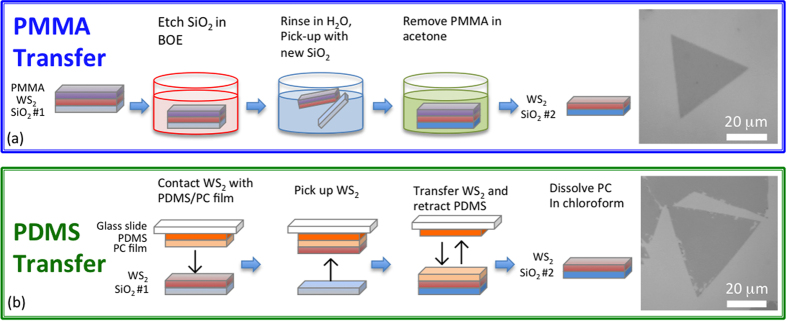
Schematic of the two transfer methods. (**a**) For PMMA transfer, the sample is coated with a thin layer of PMMA then submerged in BOE to remove the SiO_2_. Once fully etched, the film is rinsed in H_2_O then picked up with the target substrate. An acetone soak removes the PMMA. An optical image of PMMA transferred WS_2_ exhibits a uniform, clean, triangular shape. (**b**) For the PDMS transfer, a PDMS/PC film is carefully brought into contact with the desired WS_2_ then retracted. This moves the WS_2_ from Si/SiO_2_ onto the PDMS/PC film. The PDMS/PC/WS_2_ stack is then placed onto clean Si/SiO_2_. The PDMS stamp is retracted, leaving the PC film on the top surface of WS_2_, which is then dissolved in chloroform. An optical image following PDMS transfer is shown on the right.

Two distinct methods are used to remove WS_2_ monolayers from their growth substrates and transfer them to a fresh Si/SiO_2_ substrate. The first method is referred to as “PMMA transfer” and involves a wet etching process based on previous techniques used for transferring CVD graphene grown on copper^31^. This process is illustrated in Fig. 2(a) and is described in the Methods. Optical images of PMMA x-WS_2_ (Fig. 2(a), right panel) demonstrate the shape and uniformity of the flakes are maintained after transfer. Additionally, transmission electron microscope images of PMMA x-WS_2_ (Supplementary information) display a uniform, defect-free lattice composed of W and S atoms, indicating the high quality monolayer WS_2_ is preserved throughout the process. The second transfer method is free of wet etchants and ensures a clean substrate-sample interface. This method, termed “PDMS stamp”, is illustrated in Fig. 2(b) and is also described in the Methods. This technique provides microscopic precision for both placement and rotation angle of the transferred WS_2_. The optical image in Fig. 2(b) (right panel) displays a typical PDMS transferred region. The majority of the sample exhibits uniform contrast, although at the edges there are small patches where WS_2_ is absent.

**Figure 3:**
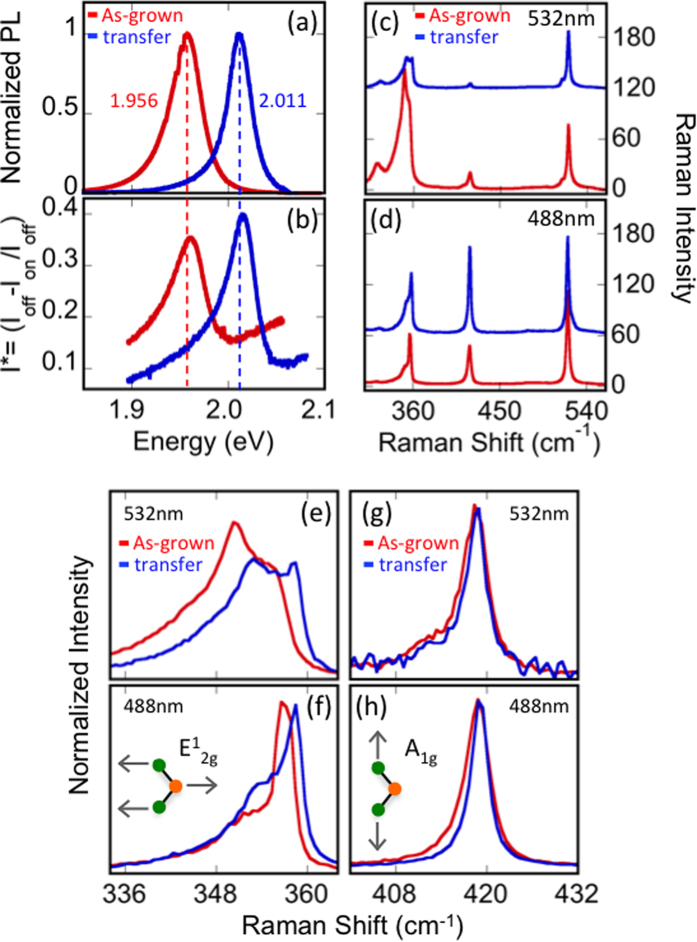
PL, reflectivity, and Raman characterization before and after PMMA transfer. (**a**) PL emission and (**b**) reflectivity shift to higher energy following transfer. A ≤ 5 meV difference in PL and reflectivity spectra is measured for both as-grown and transferred WS_2_. The vertical red and blue dashed lines indicate the peak PL position and highlight the small Stokes shift. Raman spectra are measured using (**c**) 532 nm and (**d**) 488 nm excitation. As-grown and transferred spectra are offset for clarity. The relative intensities of E^1^_2g_, A_1g_, and 2LA vary before and after. The intensity of the Si substrate peak (520.7 cm^−1^) is unaffected. (**e,f**) The transfer process shifts the in-plane Raman mode to higher wavenumber while out-of-plane (**g,h**) remains constant. Schematic diagrams of the E^1^_2g_ and A_1g_ Raman modes are displayed in the inset of (**f,h**), respectively. The changes in optical properties indicate the transfer process removes tensile strain.

PL, reflectivity and Raman spectra obtained from representative as-WS_2_ and PMMA x-WS_2_ are presented in Fig. 3. The spectrum for as-WS_2_ (Fig. 3(a)) exhibits a peak PL emission at 1.956 eV, with a FWHM of 35 meV. A considerable blue shift of ~50 meV is evident in the x-WS_2_ spectrum, where the emission peak shifts to 2.011 eV and the FWHM is reduced to 27 meV. Control samples were fabricated by coating as-WS_2_ with PMMA then removing it. These samples show no shift in peak position, ruling out doping from PMMA residue as the source of the PL shift.

In conjunction with PL measurements, reflectivity measurements are obtained from the same spot on the sample. Light from a white light source is focused though a 50× objective onto the sample and reflected intensity is measured. To minimize unwanted contributions from the substrate, the difference between the intensity measured from the bare substrate (I_off_), and from the WS_2_ sample (I_on_) is obtained then normalized to the substrate intensity, I* = (I_off_-I_on_)/I_off_. The peak reflectivity measured at 1.961 eV (2.015 eV) for as-WS_2_ (PMMA x-WS_2_) is nearly coincident with PL measurements, although there is a small difference in peak energy. This measured Stokes shift provides information regarding the doping level of TMDs, as Stokes shifts are known to increase monotonically with the doping level^32^. The small, ≤5 meV Stokes shift observed in both the as-grown and transferred samples is comparable to or less than that observed in exfoliated WS_2_ monolayers^1^,^8^, and indicates that a low doping level is achieved in as-grown material and maintained throughout the transfer process.

Raman spectra also show considerable change after PMMA transfer (Fig. 3 (c–h)). Measurements are performed with excitation energies of both 532 nm and 488 nm in an effort to reduce the resonant 2LA contribution. For both laser excitations, the relative intensities of the dominant E^1^_2g_, A_1g_, and 2LA modes show considerable differences before and after transfer. We surmise that the transfer process increases the WS_2_-to-substrate distance, leading to differences in optical interference, which in turn affects the Raman peak intensity^33^,^34^. These complicated interference effects are sensitive to both laser excitation energy as well as the energy of the Raman emission, and can thereby enhance one Raman peak while reducing the intensity of a second, as seen in Fig. 3(c,d). Therefore, it is instructive to carefully examine any alterations in peak positions (Fig. 3(e–h)).

The position of the out-of-plane A_1g_ mode in TMD monolayers is sensitive to doping level, and red-shifting occurs with increasing electron concentration, while E^1^_2g_ is unaffected by doping levels^3^,^35^. Conversely only the E^1^_2g_ mode is impacted by strain, and exhibits a red-shift as a result of uniaxial and biaxial strain^36^,^37^. For both the 532 nm and 488 nm laser excitations there is a clear ~1 cm^−1^ shift in the E^1^_2g_ peak (Fig. 3(e,f)), whereas the A_1g_ peak position is unchanged (Fig. 3(g,h)). X-ray photoelectron spectroscopy comparing as-WS_2_ and x-WS_2_ excludes changes in chemical composition as the source of the observed differences in Raman spectra (Supplementary information). Instead, the observed shift is consistent with a reduction of tensile strain^38^,^39^. The tensile strain arises due to the differences in the thermal expansion coefficient of the monolayer WS_2_ and the supporting substrate. As the substrate and WS_2_ cool to room temperature from growth temperature (e.g. 825**°** C), the two contract at different rates, imparting strain to the WS_2_ monolayer. The transfer process subsequently removes the strain. The presence of tensile strain is expected to reduce the band gap of WS_2_ at a rate of 0.2 eV per % strain^26^,^38^. We therefore estimate the strain present in as-WS_2_ on Si/SiO_2_ to be ~0.25% based on the ~50 meV shift in PL emission measured before and after transfer.

**Figure 4:**
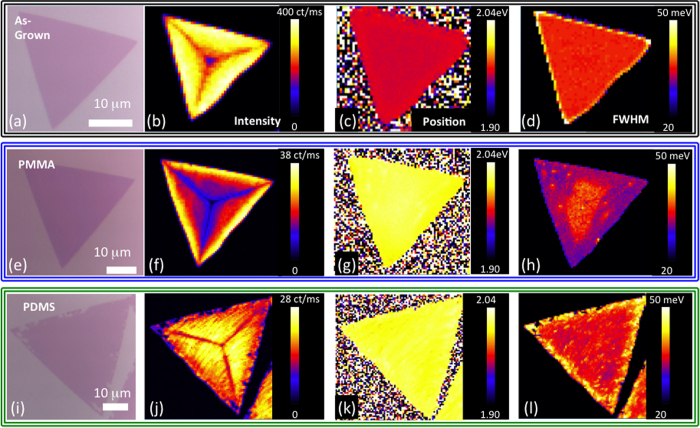
Optical images and PL maps comparing as-grown and transferred WS_2_. (**a**) Optical microscopy of as-grown WS_2_ and corresponding maps of (**b**) PL intensity, (**c**) PL emission position, and (**d**) FWHM. (**e**) Optical microscopy of PMMA transferred WS_2_ and corresponding maps of (**f**) PL intensity, (**g**) PL emission position, and (**h**) FWHM. (**i**) Optical microscopy of PDMS transferred WS_2_ and corresponding maps of (**j**) PL intensity, (**k**) PL emission position, and (**l**) FWHM. Similar patterns in PL intensity are observed for all three samples, with low intensity at the center of the triangle and directed outward toward the three corners. PMMA and PDMS transferred samples have noticeably higher emission energy, resulting from the removal of strain.

Sample uniformity is investigated across representative ~30 μm triangles. Detailed maps (Fig. 4) show the PL intensity, peak position, and FWHM for as-grown WS_2_ on Si/SiO_2_ compared to WS_2_ synthesized on Si/SiO_2_ and subsequently transferred to fresh Si/SiO_2_ using both PMMA and PDMS transfer methods. The PL emission from as-WS_2_ (Fig. 4(b–d)) exhibits considerable variation across the sample. The perimeter exhibits high PL intensity, with low intensity regions in the center that extend radially outward toward the three corners, similar to previous reports^17^,^28^. Despite the variation in PL intensity, the peak position and FWHM (Fig. 4c,d) are highly uniform across the sample, with average values of 1.964 eV and 36 meV, respectively.

The PMMA x-WS_2_ (Fig. 4(f–h)) exhibits a similar spatial pattern in PL intensity, with low intensity at the center and high intensity along the edges. Overall, the PL emission undergoes a blueshift of ~50 meV, exhibiting an average value of 2.012 eV, while the average FWHM is 32 meV. The similarity in PL intensity patterns for as-grown and transferred samples combined with the uniformity in peak position and FWHM exclude local variations in strain or doping as the cause of the non-uniform PL intensity^40^, but instead points to structural defects as the source of non-uniformity. The PL intensity of PDMS x-WS_2_ (Fig. 4(j–l)) is comparable to the PMMA transfer, with qualitatively similar PL intensity variations across the triangle, and uniform emission energy at 2.013 eV and average FWHM of 36 meV. The edges exhibit a slightly higher FWHM relative to the interior, most likely due to the tears, which are evident in the optical images. While some sample-to-sample variation is observed in emission energy following transfer, the overall shift to higher emission energy is highly repeatable, typically resulting in emission above 2.0 eV.

**Figure 5:**
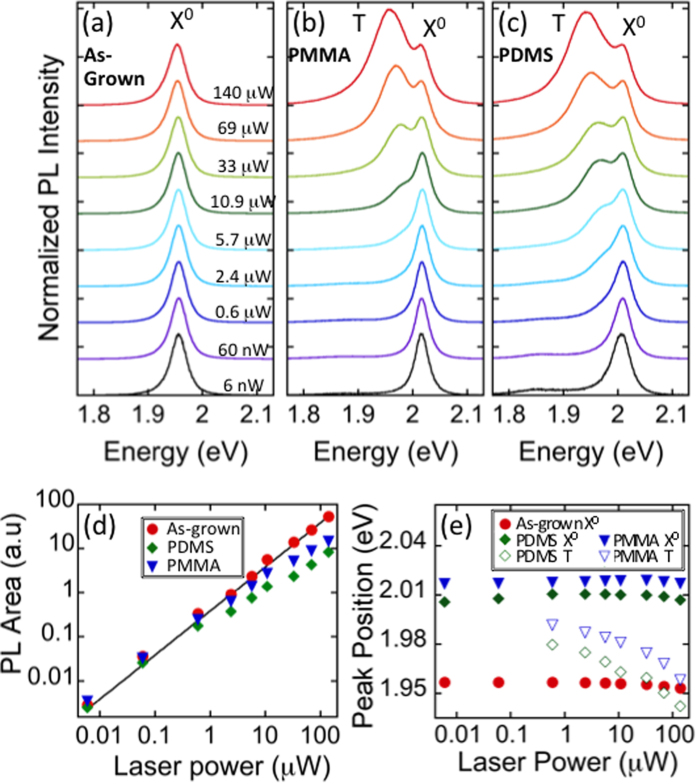
Laser power dependence in ambient conditions. (**a**) For as-grown WS_2_, the spectral shape of PL is unaffected by increased laser power, with emission occurring from the neutral exciton, X^0^, for all excitation conditions. In (**b**) PDMS and (**c**) PMMA transferred samples, X^0^ emission dominates at low powers, but the trion, T, dominates at high power excitation. (**d**) PL integrated area from 1.65 to 2.2 eV and (**e**) peak positions for the three samples.

An investigation of the relationship between emission spectra and laser excitation power is presented in Fig. 5. Recent reports have shown that exposure to a high intensity laser can enhance trionic^8^,^41^ emission as well as induce biexciton^42^,^43^ emission in WS_2_. As is evident from the normalized PL spectra of as-WS_2_ emission (Fig. 5(a)), there is a single peak for all incident powers spanning over four orders of magnitude, consistent with emission from the neutral exciton (X^0^). The integrated PL intensity is obtained within the range from 1.65 eV to 2.2 eV at each laser power (Fig. 5(d)) and is well described by a linear relationship (black line). The linearity is indicative of emission from the neutral exciton with an absence of exciton-exciton recombination. Additionally, the PL peak position of the as-WS_2_ is nearly constant, showing only a slight decrease at the highest laser powers investigated, (Fig. 5(e)) likely due to heating effects.

Transferred samples exhibit considerably different behavior under identical excitation conditions. At low power, both PMMA (Fig. 5(b)) and PDMS (Fig. 5(c)) x-WS_2_ are in the neutral excitonic state, X^0^. As laser power steadily increases, a low energy shoulder emerges then quickly develops into the dominant emission peak. The lower emission energy and larger FWHM identify the second emission peak as the charged exciton, or trion (T)^41^. The integrated PL intensity of transferred samples begins to deviate from the linear relationship above 1 μW laser power, exhibiting lower integrated area as laser power increases (Fig. 5(d)). The decrease in emission is consistent with a transfer from X^0^ to T dominated emission, as emission from charged trions is known to be lower in intensity due to increased non-radiative recombination mechanisms such as Auger recombination or exciton-exciton annihilation^3^,^8^,^44^. The position of the neutral exciton is nearly stable throughout the range of laser powers, undergoing a small redshift (<5 meV) only at the highest powers. Such behavior is similar to that observed in as-WS_2_ and suggests the heat transferred to the substrate is comparable for both as-WS_2_ and x-WS_2_. The trion in x-WS_2_, however, exhibits a clear redshift with increasing laser power (Fig. 5(e)), resulting in an increasing separation between X^0^ and T. The separation between neutral and charged excitons is linearly dependent on the Fermi level and is described by,


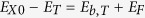


where E_X0_ is the energy position of the neutral exciton, E_T_ is the position of the trion, E_b,T_ is the trion binding energy, and E_F_ is the Fermi level^32^,^42^. The PMMA (PDMS) transferred sample exhibits a separation of 26 meV (30 meV) at the 1 μW excitation, providing an upper bound for the trion binding energy, consistent with previous results on mechanically exfoliated WS_2_^42^,^44^.

Tunability between X^0^ and T emission has previously been demonstrated in mechanically exfoliated monolayer TMDs following high powered laser exposure or annealing procedures. The likely source of the tunability is the removal or adsorption of p-type oxygen containing adsorbates^41^,^45^,^46^,^47^,^48^,^49^. We note that all our samples are measured in an ambient environment and are therefore exposed to similar concentrations of adsorbates. The stark contrast we observe between as-grown and transferred WS_2_ thus suggests very different adsorption/desorption mechanisms are present on the two types of samples. In studies of the related 2D material graphene, it has been shown that surface deformations impact sample-adsorbate interactions. Specifically, binding at local sites becomes more energetically favorable^50^. Microscopic profile fluctuations are common in mechanically exfoliated or transferred materials due to transfer-induced wrinkles or trapped chemicals^51^,^52^,^53^. This effect may be significantly reduced for as-grown materials on a rigid substrate, explaining the lack of tunability on these types of samples. Additionally, differences in sample-substrate interactions are also expected to impact adsorption behaviors. Some mechanisms include variations in strain^54^,^55^,^56^, out-of-plane relaxation at binding sites^57^,^58^, and accessibility to top and bottom surfaces^56^.

**Figure 6:**
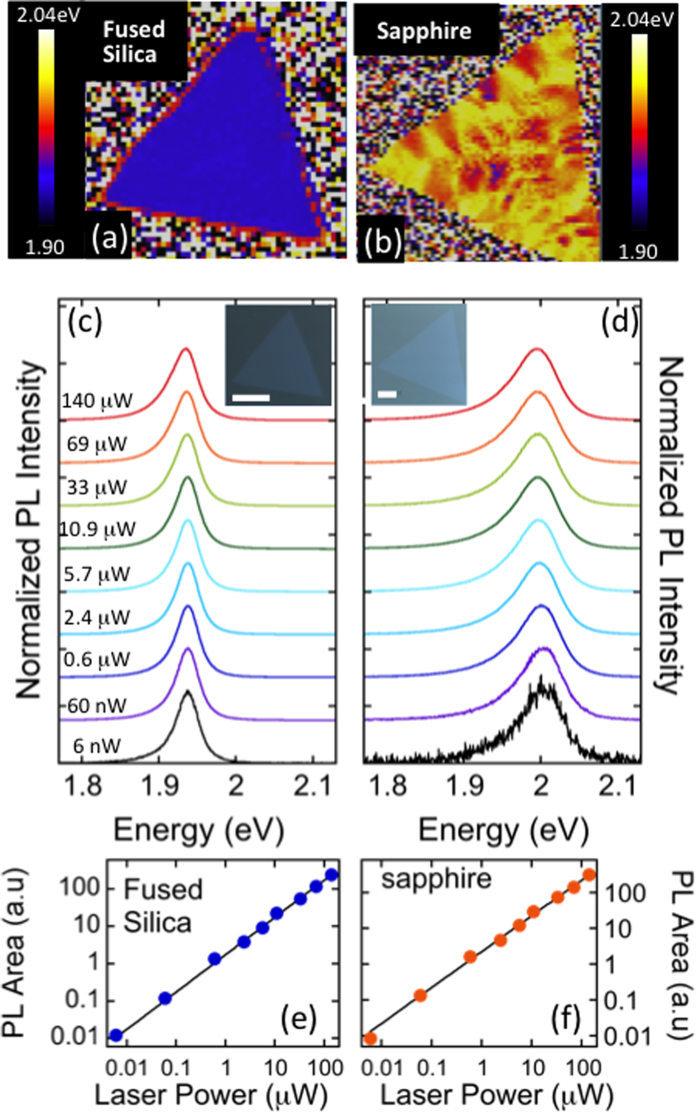
Characterization of monolayer WS_2_ synthesized on various substrates. (**a,b**) Maps of PL position for WS_2_ monolayers grown on fused silica and sapphire, respectively. The average peak position on silica (sapphire) is 1.94 ev (1.99 eV). The dissimilar thermal expansion coefficients of silica and sapphire lead to different strain in WS_2_ and the differences in peak position. The position and spectral shape of PL emission is unaffected by increased laser power for both (**c**) silica and (**d**) sapphire. (**e,f**) Integrated area is linearly related to laser power for both silica and sapphire. The insets of (**c,d**) show optical images of the mapped WS_2_ monolayers. The scale bar is 10 μm.

Synthesis of WS_2_ on substrates with different thermal expansion coefficients (α) provides a means to independently modify the strain in WS_2_ via the growth cooldown and subsequently monitor the sensitivity to laser power. Using the same procedure as reported above, we synthesize monolayer WS_2_ on fused silica and c-plane sapphire. The coefficients of thermal expansion for silica and sapphire are 0.55 and 9.7 × 10^−6^/K, respectively. The thermal expansion coefficient for WS_2_ is expected to be in the range 7 < α < 10 × 10^−6^/K based on theoretical investigations^59^ and experimentally measured bulk values^60^. As with the growth of WS_2_ on Si/SiO_2_, triangular growth is evident on both fused silica and sapphire substrates (Fig. 6). Maps of the PL peak positions are presented in Fig. 6(a,b). WS_2_ synthesized on fused silica exhibits uniform emission energy across the triangle, with an average PL peak position of 1.94 eV. In contrast, WS_2_ on sapphire results in the considerably higher average emission energy of 1.99 eV, and a large variation in peak position (1.95–2.02 eV). This large variation on sapphire could arise from factors such as substrate impurities or water intercalation^25^ and is under investigation.

The energy position of PL emission and associated Raman spectra (Supplementary information) demonstrate that synthesis on the low-α fused silica substrates induce considerable strain in WS_2_ whereas growth on sapphire approaches a strain-free system. Power-dependent PL spectra are shown in Fig. 6 (c,d) for as-grown samples on both fused silica and sapphire. Based on a value of 2.105 eV for an unstrained WS_2_ monolayer, the WS_2_ on fused silica (sapphire) exhibits ~0.39% (0.08%) strain. In both cases, the spectral shape and position are unaffected by laser power. Additionally, the integrated area of the PL emission follows a linear behavior for all laser powers used, indicating the excitonic behavior is not modified with increasing laser power. Therefore, while as-grown WS_2_ on Si/SiO_2_, sapphire, and fused silica exhibit distinctly different strain amounts, optical control of the excitonic state is absent, suggesting strain has minimal impact on adsorption and desorption mechanisms of O_2_ containing species.

## Conclusion

In conclusion, we have demonstrated that the choice of growth substrate determines the PL emission energy of the neutral exciton, X^0^. The emission energy is governed by strain in the as-grown WS_2_ and arises from differences in thermal expansion coefficient between WS_2_ and the supporting substrate. The strain can be removed by transferring the sample off of the growth substrate. This process results in the emission shifting to higher energy. We also observe a different response to laser power for as-grown versus transferred samples. Transferred samples exhibit extreme sensitivity to laser power, with X^0^ emission dominating at low laser power, but transitioning to T emission as power is increased. As-grown materials show no variation in spectral shape or position of PL as laser power is increased by more than four orders of magnitude. We speculate that the distinctly different power-dependent behaviors result primarily from different adsorption and/or desorption mechanisms in as-grown and transferred WS_2_. Differences in interfacial effects such as electron transfer between WS_2_ and supporting substrates could also influence the observed behaviors. The more intimate WS_2_-substrate contact present in as-WS_2_ may promote more efficient electron transfer from WS_2_ to the substrate, hindering trion formation. Theoretical modeling and continued experimental investigation are necessary to identify the precise source of disparity observed in as-WS_2_ and x-WS_2_.

The moderate laser powers used in this work are comparable to conditions typically utilized for optical characterization of TMDs. This highlights that care is required to prevent laser excitation-induced effects in transferred materials. Additionally, we speculate that investigations involving chemical sensing and defect passivation will be distinctly different for as-grown and transferred/exfoliated materials, as both are intimately related to adsorption and desorption mechanisms. Finally, we note this work is the first to report such dramatically different power-dependent behavior for as-grown and transferred WS_2_. This study should therefore stimulate further experimental and theoretical investigations in the field.

## Methods

### Sample preparation “PMMA transfer”

A thin layer of polymethyl methacrylate (PMMA) is spun onto the surface of the entire growth substrate then submerged in buffered oxide etchant. After several hours, the oxide layer is removed, freeing the WS_2_/PMMA film from the growth substrate. The sample is subsequently transferred to H_2_O to rinse chemical etchants, where the fresh Si/SiO_2_ is used to lift the film out of the water. A 2000 rpm spin and 150 **°**C bake improve the uniformity and adhesion to the substrate, after which the PMMA is dissolved in acetone.

### Sample preparation “PDMS transfer”

A polydimethylsiloxane (PDMS) stamp coated with polycarbonate (PC) is carefully brought into contact with the desired monolayer region. The van der Waals forces between the PC are strong enough to remove the WS_2_ from the supporting substrate. Once on the stamp, the WS_2_ can be positioned or rotated to a desired angle before being brought into contact and released onto the desired substrate with a gentle thermal anneal.

### Transmission electron microscopy

Samples are prepared for TEM imaging using the PMMA transfer process. Following the rinse in deionized H_2_O, the WS_2_/PMMA film is picked up using a Quantifoil TEM grid and subsequently baked at 150 °C on a hot plate. PMMA is removed using acetone, making the grid ready for the TEM imaging. Scanning transmission electron microscopy is performed on a Nion UltraSTEM-X 200 instrument operating at 60 kV accelerating voltage.

### X-ray Photoelectron Spectroscopy

Spectra are obtained with monochromated Al Kα radiation using a 400 μm aperture.

## Additional Information

**How to cite this article**: McCreary, K. M. *et al*. The Effect of Preparation Conditions on Raman and Photoluminescence of Monolayer WS_2_. *Sci. Rep.* **6**, 35154; doi: 10.1038/srep35154 (2016).

## Supplementary Material

Supplementary Information
