# Supplementary material for: The Effect of Preparation Conditions on Raman and Photoluminescence of Monolayer WS2
Source: Sci Rep. 2016 Oct 18;6:35154. doi: 10.1038/srep35154 (PMC5067492; doi:10.1038/srep35154)
Supplement: Supplementary Information [file srep35154-s1.pdf]

Supporting Information

Title: **The effect of preparation conditions on Raman and Photoluminescence of Monolayer WS_2**

Kathleen M. McCreary, Aubrey T. Hanbicki, Simranjeet Singh, Roland K. Kawakami, Glenn G. Jernigan, Masa Ishigami, Amy Ng, Todd H. Brintlinger, Rhonda M. Stroud, Berend T. Jonker

The photoluminescence was investigated for laser excitation in addition to 532nm. The PL intensity map acquired using laser excitation (λ_{exc}) of 488 nm exhibits clear intensity variations (Fig. S1), with lowest intensity extending from center outward to the three corners. This pattern is analogous to that obtained using 532 nm excitation (presented in the main text Fig. 4(j)) and shows the intensity variations are independent of laser excitation wavelength.

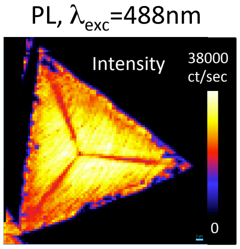

Figure S1: PL intensity map of PDMS transferred WS_2. Laser excitation of 488 nm is used. The resulting PL intensity variations are qualitatively similar to those obtained for 532 nm excitation (presented in Fig. 4j of the main text).

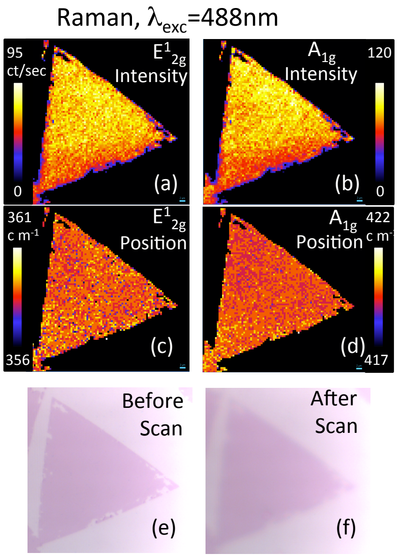

Figure S2: Raman map of PDMS transferred WS_2. Laser excitation of 488nm is used. (a,b) No discernible pattern is present in the E^1_{2g} or A_{1g} intensity, respectively. (c,d) Additionally, no pattern is present in peak position of E^1_{2g} or A_{1g}. As evident by optical images taken (e) before scanning and (f) after scanning, the sample becomes slightly out of focus during the long map. This is likely the cause of the slight intensity reduction present in the bottom third of Fig. S2 (a) and (b).

In conjunction with the photoluminescence characterization, Raman maps were acquired for λ_{exc} = 488nm. In contrast to the clear spatial variations observed in PL intensity (for both 488nm and 532nm excitation), the dominant in-plane and out-of-plane Raman peaks display no discernible pattern (Fig. S2 (a,b)). We do observe a slight decrease in overall intensity near the bottom third of the Raman maps, most likely caused by modifications to z-position of the sample. The optical images acquired before (Fig. S2(d)) and after (Fig. S2(e)) performing the Raman map show the sample has drifted away from the focal point. The observed decrease is consistent with a gradual defocussing during the course of the scan, as maps proceed from top left to bottom right. E^1_{2g} and A_{1g} peak positions (Fig. S2 (c,d)) are steady across the sample. The uniformity observed in Raman peak positions and intensities suggest structural defects (as opposed to local variations in strain or electronic doping) are the source of the observed variations in PL.

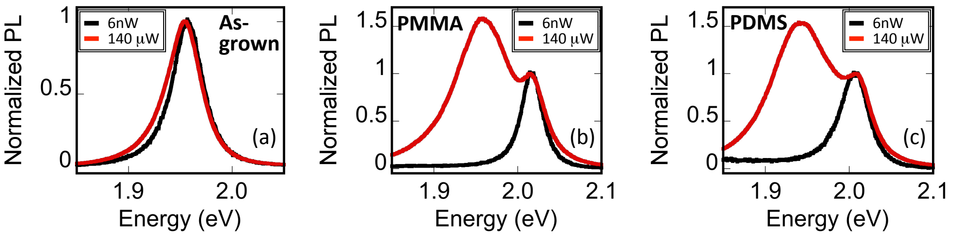

Figure S3: PL spectra acquired at low and high laser power. Spectra are normalized to the X^0 intensity. (a) As-grown WS_2 exhibits only minor differences between 6nW and 140μW excitation power. A small red-shift (~4meV) and increased FWHM is observed at higher power, most likely from sample heating. Both (b) PMMA and (c) PDMS transferred WS_2 are highly sensitive to laser power. Emission from the neutral exciton, X^0, dominates at low power, but transitions to T dominated emission with increasing laser power.

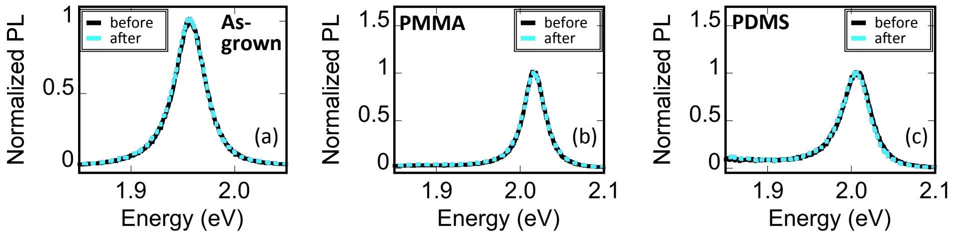

Figure S4: Comparison of PL spectra before and after power sweep. Photoluminescence is measured using 6nW laser excitation before and after exposure to 140μW laser. Nearly identical spectral shape and emission energy are obtained for (a) as-grown, (b) PMMA, and (c) PDMS samples, indicating the WS_2 samples are not damaged by laser powers utilized in this work.

Care is taken to ensure all acquisition conditions are below the damage threshold, particularly for the power-dependent investigations (presented in Fig. 5 and Fig. 6 of the main text), as high-power laser exposure is capable of damaging monolayer TMDs. Spectra in the main text are presented as the laser power is swept from low (6nW) to high (140μW). After which, the power is returned to 6nW and a final spectrum is acquired. A direct comparison of spectra obtained at low power and high power are presented in Fig. S3. Additionally, spectra

obtained for 6nW excitation are presented before and after the power sweep is completed (Fig. S4) and exhibit nearly identical spectral shape and emission position, indicating the samples are unchanged by the 140μW laser exposure.

PL and Raman spectra are measured at the same location for as-grown WS_2 on fused silica, Si/SiO_2, and c-sapphire substrates and presented in Fig. S5. As discussed in the main text, PL emission energy is sensitive to the strain in the as-grown WS_2, with increased strain resulting in a decreased band-gap and a red-shift in PL. The distinctly different emission energies indicate the largest amount of strain is present for WS_2 grown on fused silica, whereas the smallest strain is present in c-sapphire (Fig. S5 (a)). The variation in Raman E^1_{2g} peak further supports the connection between strain and growth substrate, as the position of E^1_{2g} is known to red-shift with increasing strain (Fig S5 (b)).

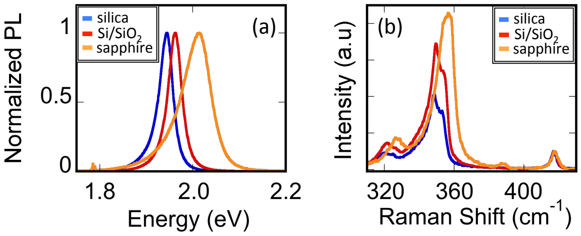

Figure S5: PL and Raman characterization of as-WS_2 synthesized on various substrates. Laser excitation of 532nm is used. (a) Photoluminescence and (b) Raman spectra are acquired at the same location for as-grown WS_2 on fused silica, Si/SiO_2, and sapphire substrates. Raman spectra are normalized to the A_{1g} peak.

In addition to the AFM image acquired on as-grown WS_2 (Fig. 1b of the main text), AFM data is obtained on PDMS and PMMA transferred WS_2. Figure S6 presents the AFM acquired from three representative PDMS x-WS_2 samples. In all three images, small particles (white spots in Fig. S6 a-c) are present on both the WS_2 and SiO_2 substrate. Such particles are not present in as-grown samples, and are most likely residues from the PC stamp and/or processing chemicals. Imperfections such as small tears (Fig. S6a) and microscopic wrinkles (Fig. S6c) are observed in some regions. The WS_2 step height for each sample is measured along the black dashed line and displayed in the inset. All three samples exhibit a step height of ~1nm, which is slightly larger than the 0.8nm measured for as-grown WS_2.

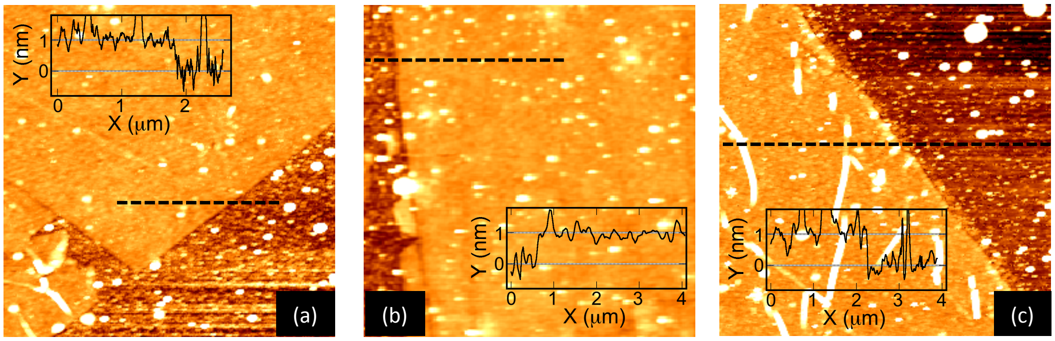

Figure S6: AFM images of PDMS transferred WS_2 on Si/SiO_2 substrate. (a-c) AFM images of representative PDMS x-WS_2. Line cuts are acquired along the dotted black line in each image and insets display the step height across the edge of the WS_2 sample. The data indicate a step height of ~1 nm for PDMS x-WS_2.

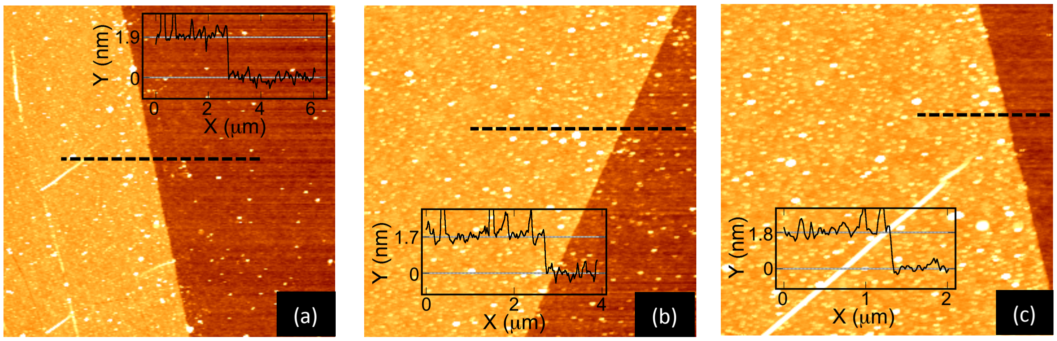

Figure S7: AFM images of PMMA transferred WS_2 on Si/SiO_2 substrate. (a-c) AFM images of representative PMMA x-WS_2. Line cuts are acquired along the dotted black line in each image and insets display the step height across the edge of the WS_2 sample. The data show variations in step heights ranging from 1.7nm to 1.9nm for PMMA x-WS_2.

The AFM acquired from several PMMA x-WS_2 samples (Fig S7 a-c) show features that are qualitatively similar to those of PDMS x-WS_2. Again, surface particles and imperfections are evident. Line cuts along the dashed line are displayed in the inset for each sample. Of note is the relatively large step height for PMMA x-WS_2, with measured values ranging from 1.7nm to 1.9nm for monolayer WS_2. Several factors may contribute to the step height value, and include effects such as increased sample-substrate distance, water layers trapped between the monolayer sample and SiO_2 substrate,[1] and the presence of PMMA residue[2] on the top WS_2 surface. Further studies are necessary to determine the exact origin of the increased step height in transferred samples. Despite the larger step height measured with

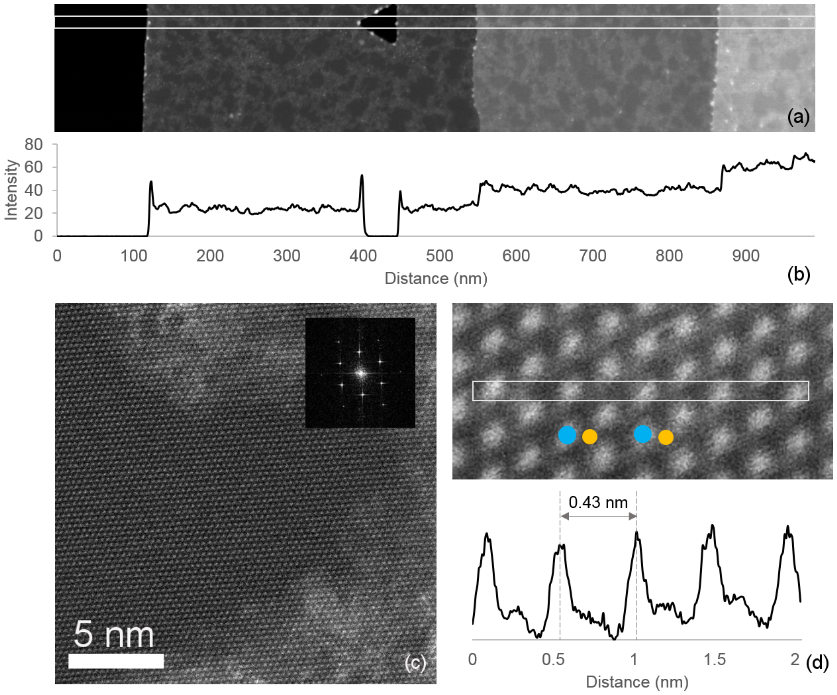

Figure S8: Scanning transmission electron microscopy of WS_2 layers. A high angular annular dark field (HAADF) image (a) of a region containing terraces of increasing thickness of WS_2 with the corresponding intensity line profile (b) displays a generally defect-free sample, where presence of multiple layers is used to calibrate and confirm the presence of monolayer WS_2. (c) Illustrates a pristine region of WS_2 with an inset of the FFT, while (d) W (light blue) and S (gold) atoms in lattice are shown with the line profile indicating the presence of both W and S atoms.

AFM for these types of samples, the modification of the Raman and PL spectra compared to the as-grown samples is the same as for the PDMS x-WS_2. Therefore, our conclusions remain unchanged, regardless of the source of the extra step height.

To assess the crystalline quality, monolayer WS_2 is imaged using high-resolution transmission electron microscopy. While the sample is predominantly monolayer, the HAADF image of a terraced region is purposefully displayed (Fig. S8 a,b) and establishes a clear intensity contrast between monolayer and multilayer WS_2. Images acquired from a single layer region exhibit a uniform, defect-free, single-crystalline hexagonal atomic structure (Fig. S8c). The measured intensity depends on the atomic number (Z) of the imaged atom as $Z^{1.64}$.[3] Therefore the bright spots correspond to tungsten atoms (Z=74) with darker contrast indicating the position of sulfur atoms (Z=16).

The chemical composition of as-grown and transferred WS_2 is analyzed using X-ray photoelectron spectroscopy. We investigate two different as-WS_2, one PDMS x-WS_2, and two separate PMMA x-WS_2 samples. All samples exhibit the same tungsten and sulfur core levels, demonstrating the chemical composition is the same for as-grown and transferred WS_2.

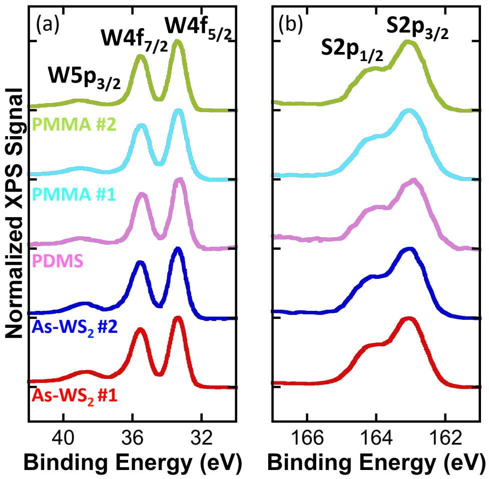

Figure S9: X-ray Photoelectron Spectroscopy of as-WS_2 and x-WS_2 samples. (a) Spectra of the tungsten core levels and (b) sulfur core levels in various as-grown and transferred samples.

References:

1. Lee, M. J. *et al*. Characteristics and effects of diffused water between graphene and a SiO2 substrate. *Nano Res*. **5,** 710–717 (2012).
2. Ishigami, M., Chen, J. H., Cullen, W. G., Fuhrer, M. S. & Williams, E. D. Atomic Structure of Graphene on SiO2. *Nano Lett*. **7,** 1643–1648 (2007).
3. Krivanek, O. L. *et al*. Atom-by-atom structural and chemical analysis by annular dark-field electron microscopy. *Nature* **464,** 571–574 (2010).
